# What in the world is global health? A conceptual analysis

**DOI:** 10.1111/dewb.12478

**Published:** 2025-02-24

**Authors:** Alberto Giubilini

**Keywords:** developing world, global South, health, health priorities, health system

## Abstract

This article suggests that the concept of global health – and to an extent the field that it designates ‐ is problematic in various ways. Within public health, the concept of the ‘public’ has been widely investigated. However, “global health” has been introduced in academic, policy, and public discussion with *comparably* lower level of conceptual, philosophical scrutiny. Thus, while public health ethics addresses the ethical and political issues that the different meanings of ‘public’ allow to identify, global health ethics tends to leave ethical and political issues raised by the concept of ‘global health’ implicit and insufficiently analysed. I will briefly present the debate around the ‘public’ in public health, describing some of the ethical and political questions that might arise, depending on what ‘public’ is taken to mean. I will then use this discussion as a conceptual map for an analogous analysis of the concept of ‘global’ in global health. I will discuss what dimensions ‘global’ adds to the concept of ‘public’. In the second part of the article, I will briefly introduce the philosophical debate on the concept of health, before suggesting that its cultural sensitivity makes it ill‐suited to be qualified as ‘global’. All in all, this article wants to bring to light the ethical implications that the terminology of ‘global health’ introduces in academic research and public policy that goes under that heading, as a first step towards better defining the ethical contours of this discipline.

## 1 INTRODUCTION

The question of what makes health ‘public’ has been the subject of much academic discussion.^1^^,^ 2 ^,^ 3 ^,^ 4 ^,^ 5 ^,^ 6 ^,^ 7 ^,^ 8 In contrast, the concept of “global health” has been introduced in academic, policy, and public discussion with *comparably* lower level of conceptual scrutiny (with notable exceptions).^9^ Efforts have been devoted to understanding it mostly from a historical, sociological, and anthropological perspective,^10^ but not so much from a conceptual philosophical perspective. Partly for this reason, the relationship between public and global health is not only complex, but also largely left unanalysed. Some suggest global health is indistinguishable from public health,^11^ and some that it is just “public health somewhere else”^12^ – more precisely, in low‐ and middle‐income countries (LMICs). Whether and to what extent these claims are true depends on what we take ‘global’ to mean. However, as I will suggest in this article, the answer is more problematic than might initially appear.

It is debatable how old the approaches and practices that came to be identified by ‘public health’ and ‘global health’ are. However, they are likely older than the terms themselves. The introduction of a term to describe pre‐existing practices is not necessarily an innocent, inconsequential move. Language often brings with it a baggage of meanings and ethical connotations that contributes both to retrospective reassessment of old practices and to reshaping them moving forward. The terms ‘public’ and ‘global’, in particular, raise questions around the complex relationships between individuals, collectives, and institutions at different levels, from small communities to the whole world. What do I owe my community? What does my community owe me? What are the boundaries of ‘my community’? And so on.

The ‘global’ framework challenges the boundaries of what we'd normally take to be ‘our community’ and invites us to think in terms of a global community, where geographical distance and cultural differences are not so relevant to the kind of obligations we have towards each other. The debate around ‘vaccine nationalism’ after COVID‐19 vaccines became available is an example. We were divided^13^ over whether rich countries ought to prioritize poorer countries^14^^,^ 15 or the interests of their own citizens^16^ in the distribution of scarce supplies of COVID‐19 vaccines. Opposition to vaccine nationalism was typically framed as a matter of ‘global health’ and global health governance’.^17^^,^ 18

The discussion about what exactly the ‘public’ in public health means is important because, depending on how one interprets the term, different ethical and political questions arise. And depending on what values one holds, different answers to those questions might follow. However, it is unclear whether and to what extent global health is concerned with the same (types of) questions.^19^ Many definitions of ‘global health’ exist, nicely captured by systematic reviews^20^ and discussed in editorials of major scientific publications.^21^^,^ 22 ^,^ 23 ^,^ 24 Yet, adequate scrutiny requires not only knowing the definitions, but understanding why terms are defined in a certain way and what makes different definitions more or less consistent with one another.

Perhaps the most widely endorsed definition is that of global health as an “area of study, research, and practice that places a priority on improving health and achieving equity in health for all people worldwide”.^25^ A lot is packed into this and other similar definitions that would require careful analysis. In fact, the use of ethics terminology (‘equity’) in this definition and the vagueness of the expression “health for all people worldwide” (according to what conception of health? What about the people who cannot be healthy?) are a good illustration of the idea that the term ‘global’ as applied to health is problematic in various ways.

This is not merely an academic discussion about terminology. In the name of global health, significant resources are mobilised, policies and programs of international aid are designed, research funding allocated, academic chairs and departments set up. An adequate level of scrutiny of the ‘global’ terminology as applied to health, and its relationship with the terminology of the ‘public’, can help clarify what types of ethical and political values we might be assuming when considering initiatives in the name of ‘global health’.

Precisely because reflection around the concept of ‘public’ in public health is relatively well developed, I will start by briefly presenting that discussion and then use it as a conceptual map for an analogous analysis of the concept of ‘global’ in ‘global health’. I will then briefly introduce the debate on the concept of health, before suggesting that it is ill‐suited to be qualified as ‘global’. I will conclude with a few examples illustrating the problems that the ‘global health’ framing might entail, if we are not aware of the risks that this article aims to emphasize.

Thus, this article wants to bring to light the ethical implications that the terminology of ‘global health’ introduces in academic research and public policy that goes under that heading, as a first step towards better defining the ethical contours of this discipline.

## 2 IN THE BEGINNING WAS PUBLIC HEALTH

### 2.1 The ‘public’ in public health

One helpful way of thinking what a concept means is by asking what it stands in contrast to. As an adjective that qualifies health, one way of thinking of ‘public’ is as in contrast with ‘private’.^26^ Some issues are a matter of private health, in the sense that they only affect me, at least in significant enough ways. These are typically protected by privacy rights or other types of individual rights (say, the right to refuse treatment). However, other issues are a matter of public health, in the sense that they also affect other people or the wider community in significant enough ways. Communicable infectious diseases typically fall in this category, although even there, the boundary between what is private and what is public is typically contested. A lot depends, of course, on what counts as significant ‘enough’ to be other people's legitimate concern, which is a contested value‐judgement. The debate on vaccine mandates is perhaps the best example of this type of negotiation.

But ‘public’ could also be taken as a noun, that is, *the* public as the entity whose health we are referring to. Again, this can be better seen if we ask what it stands in contrast to. In this sense, public health is sometimes taken to be in contrast with clinical medicine, or health care. As Tom Beauchamp and Bonnie Steinbock put it, “whereas in medicine the patient is an individual person, in public health, the ‘patient’ is the whole community or population. The goal of public health is to reduce disease and early death in populations”.^27^ In this sense, ‘public health’ is what is commonly referred to also as ‘population health’. It prioritizes approaches to health based on quantification and statistical methods^28^ within relevant groups and prevention, rather than treatment, of disease. Strategies like surveillance and data collection are central to public health policy. It comes with practical and epistemological questions about how to measure group health – which is primarily and in its original meaning a property of individuals, and only derivatively of groups.^29^ This concept of public generates its own ethical and political questions. For instance, which population is the relevant one, and how to identify it? How to account for intra‐population variations in properties and circumstances relevant for health outcomes? Is the women population, or some ethnic minority population, a distinct public for some public health purposes? And so on. This is today the most used sense of the term.^30^

Originally as well as on many current uses, however, the ‘public’ in public health refers to yet another sense of ‘public’, namely to the collective, coordinated effort required to achieve health goals, as opposed to health that is brought about by individual actions – say, a surgeon performing an operation. The required coordination can happen through the mediation of institutions, which is sometimes used to address collective action problems. One example is a government introducing a vaccine mandate to achieve herd immunity against an infectious disease.

This last sense of the ‘public’ is closely related to understandings of public health that see it as an institution itself,^31^ that is, as governance and legislation governing matters of health of the public. For Mark Rothstein, for instance, “the key element in public health is the role of the government—its power and obligation to invoke mandatory or coercive measures to eliminate a threat to the public's health”.^32^ This is the sense in which ‘public health’ has mainly been understood historically.^33^ The *Oxford Textbook of Public Health* states that “public health is concerned with the process of mobilizing local, state/provincial, national, and international resources to assure the conditions in which all people can be healthy”.^34^

Both these last two aspects – the public dimension as collective action and the public dimension as institutional action – are well captured in the early definition^35^ of the public health profession, and therefore one of the first characterizations of the professional in this field, provided by C.E.A. Winslow, namely:

Public health is the science and the art of preventing disease, prolonging life and promoting physical health and efficiency through organised community efforts for the sanitation of the environment, the control of community infections, the education of the individual in principles of personal hygiene, the organisation of medical and nursing service for the early diagnosis and preventative treatment of disease, and the development of social machinery which will ensure to every individual in the community a standard of living adequate for the maintenance of health.^36^

Importantly, while all these notions are conceptually distinct, they have historically overlapped in complex ways. For instance, public health as governance often overlaps with public health as ‘health of the public’ in that, often, the latter can more efficiently be taken care of through Governmental intervention. Similarly, public health understood in opposition to ‘private’ health has often tracked the distinction between State governance and the home, where private health matters are dealt with. Also, when talking of “the public”, especially in 18^th^ and 19^th^ Century, the concept often referred to the general population in the sense of ‘the common people’ or the ‘poor’, as opposed to elites, whose health was more often considered a personal, private matter.

On any of these understandings, the term ‘public’ is ethically and politically fertile. Each understanding raises its own distinctive ethical and political questions. For example, if ‘public’ means the opposite of private, what counts as private and what counts as public in the realm of health? If ‘public’ means the public whom health is predicated to, what criteria allow to identify the relevant public for different health‐related issues? If ‘public’ means the collective or institutional action involved in health promotion, what type of institutional intervention is justifiable – for instance in terms of coercive policies? And so on.

‘Public health’ is a source of ethical and political questions, not answers. Consider the following example. Dan Beauchamp argues^37^ that public health should become an instrument of social justice, because health is a factor that contributes to enjoyment of fair equality of opportunities. That might or might not be the case, but it is a separate ethical and political question that requires its own ethical and political analysis. For example, one might think that social justice should be pursued at a deeper, more structural level and that a good level of public health is an outcome, rather an instrument to, social justice. And different people would have different understandings of what social justice is that might translate into different public health policies, either more freedom oriented or more oriented towards public goods. This is all debatable at the ethical and political level and no doubt there would be a lot of disagreement. However, the notion of ‘public’ in public health does not itself contain the answer embedded in the very meaning of ‘public’. In principle, there can be such a thing as public health – for instance defined as the level of health of a population – without there being any obligation on society or on me to promote social justice through it. Thus, when Lawrence Gostin defines public health as “society's obligation to assure the conditions for people's health”,^38^ he has already stepped into the realm of public health *ethics*. We can disagree with Gostin about what society's obligations are while sharing with him an understanding of what public health is. Whether ‘public health’ includes any ‘obligation’, the strength of such obligations, and what these obligations precisely consist of, are ethical and political questions. In fact, they are the same ethical and political questions that arise in many other areas beyond health where we constantly negotiate the boundaries between us as individuals and us as members of the relevant ‘public’ we belong to.

The problem with ‘global’ health as a concept is that it does the opposite, that is, it is taken as a source of answers, rather than questions. More precisely, it often assumes ethical and political answers to questions that are never explicitly asked, as I am going to suggest in the next section.

## 3 THEN CAME GLOBAL HEALTH

It is unclear when the term ‘global health’ started being used. Chen and colleagues^39^ claim that the term wasn't present much in the academic literature or public discussion until US President Barack Obama's 2009 ‘Global Health Initiative’, aimed at promoting collaborations across countries to address health‐related challenges worldwide. However, they claim that “[g]lobal health as a scientific term first appeared in the literature in the 1940s”.^40^ In support of this claim, they reference a 1945 article in the *American Journal of Public Health*, titled 'Today's global frontiers in public health’. The term ‘global health’, however, did not appear in that article. Plausibly, then, the reference was simply to the fact that public health itself was for the first time seen as having no frontiers ‐ which is the same as having ‘global frontiers’.

### 3.1 The global in global health

This might be revealing of a certain way of understanding ‘global health’, that is as simply adding a geographical qualification to the ‘public’, when understood in the second sense analysed above, namely as ‘population’. On this interpretation, the qualification ‘global’ specifies that the public in question is, quite simply, that of the whole world. This is precisely the understanding of global health as ‘health for all’^41^ that became central in the global health agenda after the 1978 Declaration of Alma‐Ata and that some see as one of the defining features of global health.^42^ Historically, ‘Health *for All*’ captured both the global dimension and the dimension of the general public understood as the non‐elite. The latter is better understood by considering the context of the Cold War and socialist approaches to medicine and healthcare of that period. The former – the global ‐ is better understood not as a geographical but as a geopolitical notion.^43^ That is, it is better understood in the context of ‘international health’ and ‘international medicine’ developed by the League of Nations, “which established the concept of international health organization as a body in charge of providing guidelines and standards in health‐related matters on the basis of broad equality of people and nations”.^44^ This is the conceptual apparatus that the WHO adopted upon its foundation in 1948.^45^ Borrowing one of the conceptual distinctions I drew in the discussion of public health, we can thus take the ‘global’ to refer to both the extension of the public whose health we are talking about (the whole world population) and the governance required to deliver health interventions for that public (international organizations like the League of Nations in the first half of the 20^th^ Century and the WHO thereafter).

As Brown and colleagues note, “naming the new organization the *World* Health Organization also raised sights to a worldwide, ‘global’ perspective”.^46^ Although, as they also note, “the WHO did not invent global health”, but “other, larger forces were responsible”, including political and economic, the WHO played an important role in shaping the global health approach and agenda. It was the WHO, after all, that organized the Alma‐Ata International Conference in Primary Care, which produced the aforementioned Declaration and which is generally considered by historians of medicine “a landmark event in the history of global health”.^47^ The core message of this conference and declaration was subsequently re‐emphasized by the 2008 WHO report *Primary Health Care, Now More than Ever*. The reference, and the inspiration for the Alma‐Ata Conference, was the Primary Health Care movement that developed in the 1970s which, among other things, “urged active community participation in health care and health education at every level”.^48^ ‘Health for all the people worldwide’ is also, as we have seen in the introduction, a central element of the definition of global health with the most currency, namely, to re‐state, as an “area of study, research, and practice that places a priority on improving health and achieving equity in health for all people worldwide”.^49^

However, as a matter of fact, it is widely recognized by those working in the field^50^^,^ 51 ^,^ 52 that the focus of global health is mostly on health‐related issues in low and middle‐income countries (LMICs) – which raises concerns, among others, about colonial dynamics at play within global health.^53^ The Alma Ata conference and declaration aimed specifically to promote strategies for primary care delivery in the developing world. Historically, the focus of global health on LMICs makes sense in light of the development of ‘global health’ from the pre‐existing ‘international health’ approach, as described above. However, the terminology of ‘global’ as widely used today creates some problems at the conceptual level, with problematic ethical and political implications. Conceptually, it is unclear what makes the kinds of issues that are typically addressed within the framework of global health ‘global’ in a geographical sense, as opposed *local* public health in specific, non‐Western contexts. One would think that health issues affecting LMICs are no more global than, say, those affecting communities in suburban English areas or groups from lower socio‐economic backgrounds in London. They are public health issues that are specific to any of these areas. For instance, issues that are typically framed as global health ones such as undernutrition, malaria, or maternal mortality are heavily concentrated in sub‐Saharan Africa and Asia, and do not affect most of the Western world, so there seems to be nothing global in them.^54^ Perhaps this should not be too surprising, as it would be difficult to identify health issues that are global in the sense of affecting “all people worldwide”. Even a global pandemic like the recent COVID‐19 one impacted different parts of the world very differently, with many African countries largely unaffected by the virus itself.^55^ Addressing it in different ways in different circumstances resulted in different sets of policies and very different health outcomes. Why, then, use the ‘global’ terminology at all?

An answer might come from a second possible sense of ‘global’, that is, consistently with the taxonomy of ‘public’ provided above, as referring to the agent implementing or discussing health policies, rather than the recipient. Kayvan Bozorgmehr argues that the difficulties with different understandings of ‘global’ can be overcome by understanding it as ‘supraterritorial’.^56^ According to Andrew Lakoff, the nature of global health as an “ethically contested terrain” largely depends on ‘global’ being understood as something that “strives to transcend certain limitations posed by the national governance of public health”,^57^ and therefore something where priorities of actors and of recipients of interventions might conflict. The reference here is to the type of organization and of action taken to be necessary to address health issues *any*where (but not *every*where) in the world. The ‘supraterritorial’ dimension of global health is further emphasized by its close association with the ‘One Health’ and planetary health approaches.^58^ These focus on the health and balance of ecosystems and ultimately of the whole planet, considered essential for human health, and are also typically taken as requiring action by supraterritorial agents.

In an attempt to define global health *ethics*, David Hunter and Angus Dawson identify the geographical account of the global’ as one addressing issues that “span or sit outside of national boundaries or require global solutions”.^59^ In this sense, global health is action taken by a significant portion of the international community. In other words, ‘global’ is a qualification of what would otherwise be called ‘international health’. Consistently with the geopolitical sense of ‘global’ mentioned above, the two terms are often used interchangeably^60^ to indicate the international collaboration required to achieve certain health goals. For instance, Beaglehole and Bonita defined global health as “collaborative trans‐national research and action for promoting health for all”,^61^ a definition that would fit ‘international health’ as understood, for instance, in the context of the League of Nations mentioned above. In this sense, ‘global health’ raises questions as to whether, to what extent, and according to what principles the international community, or specific countries in it, may or should intervene in other countries.

But herein lies the problem with the ‘global’ terminology, as global’ adds to the notion of ‘international health’ a layer of ethical and political connotations. Historian Allan Brandt has pointed out that one aspect that distinguishes the notions of ‘international’ and of ‘global’ health is that the latter “is based on ethical and moral values that recognize that equity and rights are central to the larger goals of preventing and treating diseases worldwide”.^62^ Many have identified in globalization the driver of the adoption and evolution of the concept of ‘global health’.^63^^,^ 64 ^,^ 65 Bozorgmehr suggests that the scope of ‘global health’ is actually the “analysis of the ‘new’ social space created by globalization”.^66^ Historically, this might have meant that the global health approach developed as a response to globalization, and not necessarily as overlapping with it. However, once again, historical and conceptual analysis raise different issues. Conceptually, the ‘global’ terminology is today linked with the widespread globalization mindset more generally, which, again, has ethical and political implications. Let's see in more detail.

Above, I asked “why, then, use the ‘global’ terminology at all”, if it doesn't reflect the fact that the field is concerned with local issues in specific contexts? One possible reply is that it is simply a label and, as such, we should not read too much into it. It might be conceptually problematic, but, as labels often do, it serves well pragmatic purposes without having to *explain* what it refers to. For instance, rhetorically it could be helpful for fundraising purposes or for raising awareness of some specific health issues affecting particular areas of the world. However, the second sense of ‘global’ illustrates why ‘global’, far from being an innocent, neutral label, is actually a problematic one. There are important ethical and political questions about responsibilities towards the health of local communities “somewhere else”^67^ or “outside national boundaries”^68^ that depend on ethical and political aspects of globalization. First and foremost, as put by King and Koski, “[m]ost communities have pressing health problems at home; why go somewhere else?”^69^ This is not a rhetorical question. Whether and how to intervene, who should intervene, and who should decide whether to intervene, all require some ethical and political justification. The answers depend on a set of ethical and political values, particularly around equity and our obligations towards our own communities *vs* obligations to communities living far away. These values guide societal, political, and personal choices that are broader than just health. They are subject to different interpretations and political disagreement, for instance when discussing what percentage of a country’ gross national income should be destined to foreign aid. It is therefore important that the concept ‘global’ as applied to health does not implicitly introduce values and answers to these questions that are simply assumed, rather than critically examined. Unfortunately, the ‘global’ terminology comes precisely with that risk.

A recent systematic review of definitions of ‘global health’ found that “equity and social justice were the two most commonly and explicitly referenced values undergirding [global health] definitions and goals. Equity was repeatedly framed as a ‘main objective’ and core component of [global health] research and practice”.^70^ This is true of academic approaches and governance alike. Thus, for instance, regarding the former, the Consortium of Universities for Global Health defines global health as “a field of study, research and practice that places a priority on achieving equity in health for all people”; and regarding the latter, the UK Health and Security Agency says that “global health is focused on improving health and achieving health equity for all people worldwide – meaning working towards the absence of avoidable, unfair, or remediable differences among groups of people”.^71^ Koplan's and colleagues’ canonical definition of public health cited above as an “‘area of study, research, and practice that places a priority on improving health and achieving equity in health for all people worldwide” shares this aspect.

This conception of ‘global health’ can explain why the discipline almost exclusively focuses on LMICs and does not consider, say, public health in equally ‘global’ (or equally ‘local’) contexts, such as suburban or rural areas within Great Britain or the US, also characterized by significant health disparities.^72^

So why go somewhere else? From within the global health framework, the answer is straightforward and not up for debate: because that is what equity and social justice applied on a global scale require, according to the values already built into the definition of ‘global health”. Some accounts of ‘global health’ explicitly frame it in terms of ‘globalisation of public health’,^73^ but from what I have said so far, that approach is often implicit in the notion of global health. If ‘global’ is taken to mean something morally loaded with the kind of values typically associated with globalization, cosmopolitanism, and the idea of a ‘global community”,^74^ whereby our obligations to others extend beyond kith and kin, then the framework incorporates answers to ethical and political questions into the definitions. In this way, disagreements on how to interpret those ethical and political values cannot represent dissenting voices within the ‘global’ framework. Instead, they are ground for excluding such dissenting voices from that framework. That is, by definition, the ‘global’ in global health incorporates the commitment to very substantive ethical and political values regarding cosmopolitanism and globalization around which, however, reasonable disagreement in society persists – most obviously, not everyone agrees with globalization and, among those who do, not everyone agrees on what globalization should entail.

We saw above that ‘public health’ is a fertile concept because the notion of ‘public’ generates different ethical and political questions, depending on how it is understood. On the contrary, the concept of ‘global’ in ‘global health’ seems to provide answers that haven't been properly scrutinized about ethical and political questions that have been bypassed. For when seen from outside of the ‘global’ framework, what being ‘fair’ and ‘equitable’ means is a complex matter. For instance, what level of health disparity across the world does equity require to address? Is the cosmopolitan^75^ interpretation of equity centred on equal consideration for equal interests of all, regardless of geographical distance,^76^ preferable to a ‘sufficientarian’ one which only requires that the worst off are brought up to a minimum level of wellness, regardless of whether inequalities persist? And what is an ethically acceptable minimum, and according to what criteria?

If substantive notions of fairness and equity are built into the definition of global health, then those who disagree with that cosmopolitan ethos are automatically excluded from the field. They are transformed into “enemies” of the discipline, or even, according to some, “racist” opponents.^77^ In this way, the discipline makes itself immune from internal ethical and political questioning by excluding through stipulative definitions those who would want to challenge its underlying ethos.

Consider the debate around vaccine nationalism during the recent pandemic. Disagreement around it is centred on ethical and political disagreement over duties to one's community *vs* duties to the international community. The ‘global’ framework of ‘global’ health and the values of equity and fairness built into the definitions already presuppose moral opposition to vaccine nationalism. In fact, a lot of criticisms of vaccine nationalism during the recent pandemic was raised in the name of global health^78^ and therefore as a matter of “global health governance”.^79^ But what if ‘vaccine nationalism’ is, at least in part and on some version of it, ethically justifiable? Many would think governments do have a duty to prioritize their own citizens’ interest when it comes to distribution of health resources,^80^ and some advocate for middle ground positions.^81^ Any of these views is, of course, questionable, but the point is precisely that there are questions to be asked. The morally loaded terminology of ‘global health’ reflected in most definitions seems to already presuppose the answer or, at least, seems to fail to acknowledge that there are questions to be asked.

Those leaning towards vaccine nationalism do not think that vaccine nationalism is inequitable or unfair. Instead, more plausibly, they might think that what is equitable and therefore fair is that a Government gives a certain degree of priority to its own citizens. So there is an underlying disagreement on how fairness is to be understood in the context of Government obligations. By defining global health in terms of equitable approach and assuming that equitable approach means prioritizing countries on the basis of needs, one is already assuming a certain substantive ethical and political view by definition, rather than via the means of ethical and political discussion.

## 4 HEALTH, THE PUBLIC, AND THE GLOBAL

Does the fact that health is qualified as public or global affect the way we think of it? This is the question I will be concerned with in this section. It is useful to start with a brief overview of the debate around the concept of health in the philosophy of medicine. The debate is too complex to give an adequate account here. I will, however, present a short, simplified version of it to give an idea of the inevitably cultural and political dimension constitutive of any conception of health, before examining the implications of this for the concepts of public and global health.

### 4.1 Health

The two paradigmatic accounts of health discussed in the philosophy of medicine are the ‘naturalistic model^82^ and the ‘normative model”,^83^^,^ 84 which are sometimes integrated into hybrid models. There are also sceptical accounts according to which there can be no unified explanation of what health is, considering the complexity of its biological, normative, political, and phenomenological dimensions.^85^

Christopher Boorse^86^ thought that his biostatistical model of disease and health –the canonical example of the first type – had the advantage of being value‐neutral because based on the objective measurement of certain specific physiological parameters. On his view, health in an individual is the statistically normal contribution of the individual's parts to the individual's survival or reproduction, where statistical normality is defined on the basis of the individual's relevant reference class. Reference classes are natural kinds: age, sex, and species. This stands in contrast to normative accounts, that is those cashed out mainly in terms of states that it would be ‘good’ (health) or ‘bad’ (disease) to have.^87^ Values are here embedded in the definition itself. The WHO's definition of health in terms of complete physical, psychological, and social wellbeing is normative because it equates health with something considered valuable – namely, wellbeing. On this view, a dysfunction that does not translate into loss of wellbeing is not a diseased state because nothing of value is lost.

Hybrid models have also been proposed.^88^^,^ 89 ^,^ 90 ^,^ 91 On hybrid accounts, a biological dysfunction is necessary but not sufficient for something to count as a disease, as it needs to be accompanied by some (properly) disvalued state. Some have argued that, ultimately, there inevitably is a normative element in naturalist accounts^92^^,^ 93 and a naturalist element in normative accounts.^94^ Perhaps, then, any account of health is inevitably hybrid. For instance, some have suggested that the biostatistical model “is not a naturalist conception of disease” because “in selecting age, sex, and species type, Boorse is making value judgments regarding the saliency of particular properties to determine the members of each reference class”.^95^

Boorse has offered replies to these objections,^96^ whose assessment is beyond the scope of this paper. Here, I want to focus on a different, more fundamental source of normativity of the definition of health. That is the semantics itself of the concept of health. Health is, *by definition*, something good, worth pursuing, and perhaps that we have some obligation to promote in some way, however else we want to conceptualize it. Regardless of how else we conceptualize it, any understanding of health needs to start from the presupposition, which is part of the meaning itself of the term, that health is “of intrinsic value to human beings as well as being instrumental for other components of wellbeing”.^97^ If health is defined in terms of wellbeing, that is because wellbeing is considered valuable. If it is defined in terms of normal functioning, that is because normal functioning is considered valuable.^98^ An organism characterized by biostatistically normal functioning would not be called ‘healthy’ if we didn't think that statistical normality is worth pursuing. Similarly, we would not think that the functioning that contributes to survival and reproduction is valuable, and therefore expresses a healthy status, if we didn't consider survival and reproduction valuable and indeed more valuable than other goals.^99^ Some would go as far as to say that health is what philosophers of language would call a ‘thick concept’,^100^ that is, something whose meaning cannot be fully understood without the positive connotation associated with it (like the words ‘virtue’ or ‘courage’, which describe some properties but also at the same time, *by definition*, express a positive evaluation of them).

The reliance of the conception of health on values is even more marked in the context of public health discussion. Traditional discussion of what health is within the philosophy of medicine has focused on health as a property of individuals, and therefore with a primary focus on clinical medicine. An analysis of the concepts of health and disease within the context of public health adds layers of complexity tied to its ethical and political dimension.^101^

### 4.2 Health and the public

All the conceptions of health analysed so far are attributes of individuals, not of populations. It is always an individual, and never a group, that can be identified as having a pathological state on these accounts – whether the naturalistic element is understood in biostatistical terms^102^ or in evolutionary terms.^103^^,^ 104 These accounts give rise to conceptual problems about how to understand and assess the health of a group.

An individual can be healthy or unhealthy, or more or less healthy, depending on how his/her parts overall function or how he/she overall feels. But a collective? It is unclear in what sense it can be said that a group is healthy, except as a function of the health of individuals scaled up according to some factor (aggregation, average, and so on). And a solid philosophical conceptual framework for ‘group wellbeing’ is lacking.^105^

Some have attempted to formulate a conception of health that applies directly to collectives, rather than to individuals. Morar and Skorburg, for instance, provide a “hypothesis of extended health” whereby the bearer of health doesn't need to be an individual. As they claim, “certain features of our biological and social environment can be so tightly integrated as to constitute a unit of care”, so that “the bearers of health and disease states are these dynamic functional units”.^106^ Similarly, Smart^107^ draws on analogy between human groups and bee colonies to conceptualize health as applicable to human populations, on the assumption that “human societies are not unlike bee colonies” in relying on coordination among individual members each fulfilling their ‘functional role’ in the attainment of natural goals. He postulates that in the same way as the interconnectedness of the bees allows to say that a bee colony, as a unit, is healthy or diseased, so the interconnectedness of humans within societies qualifies a human group, as a unity, for health status, independently of the health status of its individuals. This account is, however, problematic, for two reasons. First, there obviously are many relevant ways in which human societies are different from bee colonies, largely due to the many relevant ways in which humans are different from bees – for instance, in having ethical and political values to regulate societal coordination, which explains why societal functioning is not homogenous across different human societies in the way it is across different bee colonies. Second, and more importantly for the purpose of this article, the ‘health’ referred to in these analogies is a different notion of health from the one normally referred to in discussion of public health. The concept of health here deployed seems metaphorical, or, at best, it would be synonymous of ‘well‐functioning’. But that would be no more meaningful than saying than any well‐functioning system, whether human or of any other kind, is ‘healthy’ (for instance, my car is healthy in the same sense, but that is not a very meaningful notion of health).

If we want to apply a meaningful notion of health to a ‘public’, then we need to ask conceptual and ethical questions. For instance, how many individuals, and which individuals, need to be healthy for a public to be healthy (conceptual question)? And how many, and which ones, for the level of public health to be considered good (ethical question)? One might think of the health of the public in an aggregative sense, whereby the more individuals there are who are healthy, the healthier the public is. This might answer the conceptual question, but not necessarily the ethical one, as it is insensitive to individual variations and health inequalities within a population. So one might suggest that the relevant indicator – either at the conceptual or at the ethical level ‐ is the average level of health of a population, so that what matters is not so much how many healthy individuals there are, but how healthy the average person is. However, averages are insensitive to how badly off the worse off members of the relevant population are. So one might propose a *maximin* criterion or a sufficientarian criterion, whereby the health of a population is determined by how badly off the worse off members are. And so on.

These problems are just a few examples that illustrate how the concept of public health is conceptually complex and ethically fertile. The notion of “public health” is, in this sense, the starting point of conceptual, ethical, and political discussion.

### 4.3 Health and the global

The analysis I have just provided of health becoming public can be replicated in the case of health becoming global. The conceptual and ethical problems about determining and assessing the health of the public are scaled up. Quantifying the health of the ‘global’ community – either by aggregation, or by averaging, or by any other method – means quantifying over a range of values and conceptions of health that might be not only difficult to identify, but indeed incommensurable, given the value‐laden nature of the concept of health. That is because the relevant public is not only a heterogenous group of individuals, but also a heterogenous group of communities with marked cultural, political, demographic, and socioeconomic differences, as well as, of course, individual variations within those communities. All these can inform in different ways local conceptions and assessments of health.

Once again, the risk is that the ‘global’ terminology presupposes uniformity on final goals, ways of pursuing them, and ethical and political values. As King and Koski^108^ put it, “global health interventions often implicitly assume an expertise gradient, in which we—a ‘we’ that is drawn almost exclusively from wealthy countries in the Global North— have superior understanding about how best to identify, prioritise and solve pressing health problems somewhere else”.

The ethical and political values involved in our understanding of health are inevitably context sensitive.^109^^,^ 110 It is true that the value‐laden and context‐sensitive nature of the concepts of health and disease would not allow for much variation in certain cases –diabetes, for instance, seems to be a clear case of disease, no matter the cultural context. However, such value‐laden and context‐sensitive nature would entail different approaches to conceptualizing, pathologizing, preventing, treating certain disease states. Although diabetes would likely be considered a diseased state in pretty much every cultural context, it has no uniform characteristics, no clear causes, and a variety of symptoms which could be addressed in different ways. Different approaches might prioritize adjustments in lifestyle choices over pharmacological interventions. Indeed, they might not consider it a *uniform* disease category.^111^^,^ 112 All these factors can explain why both the categorization of the disease and the perception of it might differ from context to context. For instance, in sub‐Saharan Africa unique types of diabetes have been identified, such as what some call “malnutrition‐modulated diabetes mellitus”, whose nature and indeed whose very existence are contested ‐ Kenyan diabetologist Tom Johnson described it as ‘a syndrome seeking clarity’. And in the same area, the perception of diabetes is affected by “cultural factors and health beliefs” whereby “under‐nutrition and opulence coexist; food remains a daily challenge and overweight can be subsequently perceived as a sign of wealth. Indeed, being obese is a deeply rooted status symbol”.^113^ The perception of diabetes, its causes, and its symptoms are therefore subject to different evaluative judgments, including some with positive connotations deriving from local cultural influences on the perception of obesity. Thus, diabetes can be conceptualized differently, perceived differently, and treated differently depending on such context‐sensitive factors. For instance, in Sub‐Saharian Africa it has been suggested that the involvement of “traditional healers should be considered as part of the treatment options", given that “to the everyday African, they are very much a part of illness management”.^114^ But other cultural contexts would have their own specific ways of approaching it. The typically ‘global’ nature of diabetes doesn't account for the microhistories of the disease and living with the disease in a particular LMIC country (see e.g. the work by Moran‐Thomas for an account of the experience of living with diabetes in post‐colonial Belize).^115^

Also, the relative priorities of treating certain diseases and of other valuable aspects of public health can vary greatly across cultural contexts. For example, an ‘Afro‐communitarian’ ethics framework^116^ centred on the values of interconnectedness and interdependence of all beings^117^^,^ 118 prioritizes relationality and therefore community interests over individualistic pursuit of wellbeing, more typical of Western contexts. These differences in values establish differences in the way health is conceptualized and different health goals prioritized in different contexts. In some cultures, one's own wellbeing is considered interdependent with the wellbeing of others in one's community,^119^ as opposed to something that individuals can pursue by themselves once they have, individually, access to the relevant medical technology.^120^

The global health framework risks disregarding the context‐sensitivity of local normative frameworks for health and implement interventions in the name of a concept of health and of health care not locally shared.^121^ As such, global health risks becoming an instrument of what Barnett and Duvall have called “productive power”, that is, power in the creation of meanings through the use of concepts – such as health and global health ‐ that force us to think of the world in some ways, but not others.^122^

## 5 CONCLUSION

Jeremy Shiffman^123^ has argued that in global health we see individuals and organizations who “create concepts for thinking about health priority‐setting, such as burden of disease, treatment cost‐effectiveness and the right to receive care”. The problem is that the individuals and organizations that create the relevant concepts – for instance, understandings of fairness or equity ‐ in the name of global health do not always capture the interests, needs, and complexities of the communities that they are aimed at. This underlying tension might well reflect different priorities, rather than different ways of working towards the same goal. Andrew Lakoff has helpfully identified two main types of ethical ‘regimes’ that characterize global health. One – global health security focused on ‘emerging infectious diseases’ – prioritizes protecting wealthy countries from infectious disease threats that might originate in developing countries, but that don't exist yet. The other one – ‘humanitarian biomedicine’ – targets diseases that affect mostly poor countries, aimed at alleviating the suffering “independently of national and social identity”.^124^ These are clearly two different types of ethical goals of global health action, but the difference between the two risks being overlooked if the ‘global’ terminology is used without adequate scrutiny of its implicit ethical assumptions.

According to Lakoff, “the type of ethical relationship implied by a project of global health depends on the regime in which the question is posed: the connection between health advocates and the afflicted (or potentially afflicted) can be one of either moral obligation to the other or protection against risk to the self. Global health is, in this sense, a contested ethical, political and technical zone whose contours are still under construction”.^125^ The analysis provided in this paper wants to be a step, if not towards the construction of the contours of global health as an area of inquiry and of action, at least towards a better understanding of what is at stake when the ethical contours have to be drawn.

The case of the COVAX initiative during the recent pandemic is a good illustration of Lakoff's point and of some the problems with the notion of ‘global health’ I have discussed in this article. In the name of global equity, as a matter of “global health governance”, and in response to vaccine nationalism,^126^ COVAX was set up and heavily funded via the WHO. Africa has been relatively mildly affected by COVID‐19 compared to other public health issues that affect many African countries, including malaria, and also compared to the rest of the world.^127^ Yet, three times as much was spent in one year to deliver COVID‐19 vaccines than for malaria treatment in Africa.^128^ This type of initiatives was often accompanied by the slogan “we are all in it together”, but again, it is worth asking whether this type of slogan is part of the same ‘global’ approach that is insensitive to differences in needs and priorities.

Indeed, systematic reallocation of large‐scale health resources in the name of a ‘bigger risk’, leaving an otherwise larger but local threat unattended to, is well‐documented historically. For instance, in colonial India some diseases like the bubonic plague and cholera saw an immense allocation of resources because they affected trade, diplomatic relations, and resulted in international pressure. However, the ‘true plague’ of India was malaria, which saw comparatively low resource allocation, since it was endemic and therefore relatively invisible to the international community, did not affect trade, and thus was not really an international or ‘global’ problem.^129^

These are just a couple of examples of the risks implied by a notion of 'global health' which too often carries with it assumptions about the global, about health, and about what it means for health to be global. In this article, I have tried to unpack some such assumption by problematizing the very notion of “global health”. Some take the new WHO Pandemic Agreement to repropose this type of problems, particularly by neglecting the specificity of local contexts, and call for the decolonisation of global health.^130^^,^ 131 Whether ‘decolonising’ is the right framework and the right strategy is a contested,^132^ complex^133^ matter that I am here happy to leave open. Rather than provide any solution, this article more simply tried to diagnose a problem with the notion, and the approach, of ‘global health’.

To be sure, this article is not necessarily a call for dropping the ‘global’ terminology. Global health does not need to present the problems I have brought to light in this article. For instance, one could argue that the ‘local’ should inform the ‘global’ and the global doesn't need to homogenise populations and places. Examples of initiatives in this direction exist, such as the ‘Local is Global’ doctrine promoted by the Global Health Institute at Duke or, more generally, the notion of ‘glocalization’ of public health.^134^^,^ 135 We could argue for a ‘global health’ that is culturally sensitive and geographically diverse. That is desirable, but it is something that can only be achieved if we become aware of the problematic implications that the terminology of ‘global health’ is introducing in academic research and public policy that goes under that heading.

## CONFLICT OF INTEREST STATEMENT

None to declare.

## OPEN ACCESS

For the purpose of Open Access, the author has applied a CC BY public copyright licence to any Author Accepted Manuscript (AAM) version arising from this submission.

## Footnotes

1 Beauchamp, D. E., & B. Steinbock. (Eds.). (1999). *New Ethics for the Public's Health*. New York: Oxford University Press.

2 Rose, G., Khaw, K. T., & Marmot, M. (2008). *Rose's Strategy of Preventive Medicine*. New York: Oxford University Press.

3 Rickles, D. (2011). Public Health. In F. Gifford (Ed.), *Handbook of the Philosophy of Science (pp.523‐572)*. Volume 16: *Philosophy of Medicine*. Elsevier BV.

4 Verweij, M., & Dawson, A. (2007). The Meaning of ‘Public’ in Public Health. In A. Dawson & M. Verweij, (Eds.), *Ethics, Prevention and Public Health* (pp.13‐29). Oxford: Oxford University Press.

5 Jennings, B. (2007). Public Health and Civic Republicanism: Toward an Alternative Framework for Public Health Ethics. In A. Dawson (ed.), *Ethics, Prevention, and Public Health* (pp. 30‐58). Oxford: Oxford University Press.

6 Coggon, J. (2012). *What Makes Health Public? A Critical Evaluation of Moral, Legal, and Political Claims in Public Health*. Cambridge University Press.

7 Faden, R., Bernstein, J., & Shebaya, S. (2022). Public Health Ethics. In E. N. Zalta (Ed.), *The Stanford Encyclopedia of Philosophy*. Metaphysics Research Lab, Stanford University.

8 John, S. (2022). Groups and individuals. In S. Venkatapuran & A. Broadbent (Eds.), *The Routledge Handbook of Philosophy of Public Health* (pp. 40‐52). Routledge.

9 Bozorgmehr, K. (2010). Rethinking the ‘global’ in global health: A dialectic approach. *Global Health*, 6, 19.

10 E.g. Farmer, P., Kleinman, A., Kim, J., & Basilico, M. (Eds.). (2013). *Reimagining Global Health: An Introduction*. University of California Press.

11 Fried, L. P., Bentley, M. E., Buekens, P., Burke, D. S., Frenk, J. J., Klag, M. J., & Spencer, H. C. (2010). Global health is public health. *Lancet*, 375(9714), 535‐537.

12 King, N. B., & Koski, A. (2020). Defining global health as public health somewhere else. *BMJ Global Health*, 5(1), e002172.

13 Vanderslott, S., Emary, K., te Water Naude, R., English, M., Thomas, T., Patrick‐Smith, M., Henry, J., Douglas, N., Moore, M., Stuart, A., Hodgson, S. H., & Pollard, A. J. (2021). Vaccine nationalism and internationalism: Perspectives of COVID‐19 vaccine trial participants in the United Kingdom. *BMJ Global Health*, 6(10), e006305.

14 Hussain, M., Sadigh, M., Sadigh, M., Rastegar, A., & Sewankambo, N. (2023). Colonization and decolonization of global health: Which way forward? *Global Health Action*, 16(1), 2186575.

15 Schuklenk, U. (2021). Vaccine nationalism – at this point in the COVID‐19 pandemic: Unjustifiable. *Developing World Bioethics*, 21(3), 99.

16 Ferguson, K., & Caplan, A. (2021). Love thy neighbour? Allocating vaccines in a world of competing obligations. *Journal of Medical Ethics*, 47(12), medethics‐2020‐106887.

17 Pushkaran, A., Chattu, V. K., & Narayanan, P. (2023). A critical analysis of COVAX alliance and corresponding global health governance and policy issues: A scoping review. *BMJ Global Health*, 8(10), e012168.

18 Schuklenk, op. cit. note 15.

19 Benatar, S., & Upshur, R. (2011). What is global health? In S. Benatar & G. Brock (Eds.), *Global Health and Global Health Ethics* (pp.13‐23). Cambridge University Press.

20 Salm, M., Ali, M., Minihane, M., & Conrad, P. (2021). Defining global health: findings from a systematic review and thematic analysis of the literature. *BMJ Global Health*, 6(6), e005292.

21 Horton, R. (2014). Offline: The case against global health. *The Lancet*, 383(9930), 1705.

22 Horton, R. (2023). Offline: The case for global health. *The Lancet*, 401(10389), 1639.

23 Beaglehole, R., & Bonita, R. (2010). What is global health? *Global Health Action*, 3:10.3402/gha.v3i0.5142.

24 Chen, X., Li, H., Lucero‐Prisno III, D. E., Abdullah, A. S., Huang, J., Laurence, C., et al. (2019). What is global health? Key concepts and clarification of misperceptions. *Global Health Research and Policy*, 5, 14.

25 Koplan, J. P., Bond, T. C., Merson, M. H., Reddy, K. S., Rodriguez, M. H., Sewankambo, N. K., & Wasserheit, J. N. (2009). Towards a common definition of global health. *The Lancet*, 373(9679), 1993‐1995.

26 Coggon, op. cit. note 6, esp. pp. 31‐42.

27 Beauchamp & Steinbock, op. cit. note 1, p. 25.

28 Rickles, op. cit. note 3.

29 Ibid.

30 Coggon, op. cit. note 6.

31 E.g. Acheson, D. (1988). Report of the Committee of Inquiry into the future development of the public health function. London: HMSO; Public Health in England.

32 Rothstein, M. A. (2012). The future of public health ethics. *American Journal of Public Health*, 102(1), 9.

33 Berridge, V. (2016). *Public Health: A Very Short Introduction*. Oxford University Press.

34 Detels, Roger, and Breslow L. (2021). Current scope and concerns in public health. In R. Detels, J. McEwen, R. Beaglehole, & H. Tanaka (Eds.), O*xford Textbook of Public Health* (pp. 3–20). Oxford: Oxford University Press.

35 Berridge, op. cit. note 33.

36 As cited in Berridge, op. cit. note 33.

37 Beauchamp, D. E. (1976). Public Health as Social Justice. *Inquiry*, 13(1), 3–14.

38 Gostin, L. O. (2001). Public health, ethics, and human rights: A tribute to the late Jonathan Mann. *The Journal of Law, Medicine and Ethics*, 29(2), 121–130.

39 Chen, et al., op. cit. note 24.

40 Ibid:14.

41 Gostin, L. O. (2012). A framework convention on global health: Health for all, justice for all. *JAMA*, 307(19), 2087–2092.

42 Beaglehole & Bonita, op. cit. note 23.

43 Brown, T., & Moon, G. (2012). Geography and global health. *The Geographical Journal*, 178(1), 13–17.

44 Borowy, I. (2011). International health work: The beginnings. *Michael*, 8, 210–221.

45 Brown, T. M., Cueto, M., & Fee, E. (2006). The World Health Organization and the transition from “international” to “global” public health. *American Journal of Public Health*, 96(1), 62–72.

46 Ibid:64.

47 Basilico, M., Weigel, J., Motgi, A., Bor, J., & Keshavjee, S. (2013). Health for All? Competing Theories and Geopolitics. In P. Farmer, J. Y. Kim, A. Kleinman, & M. Basilico (Eds.), *Reimagining Global Health: An Introduction*. (pp. 74–110). University of California Press, p. 74.

48 Brown, et al., op. cit. note 45, p. 64.

49 Koplan, op. cit. note 25, p. 1993

50 King & Koski, op. cit. note 12.

51 Smeeth, L., & Kyobutungi, C. (2023). Reclaiming global health. *The Lancet*, 401(10377), 625‐627.

52 Horton, op. cit. note 22.

53 Greene, G., Thorp Basilico, M., Kim. H., & Farmer, P. (2013) Colonial Medicine and Its Legacies. In P. Farmer, J. Y. Kim, A. Kleinman, & Basilico, M. (Eds.), *Reimagining Global Health: An Introduction* (pp. 33‐73). University of California Press.

54 Bozorgmehr, op. cit, note 9.

55 Osayomi, T., Adeleke, R., Akpoterai, L. E., Fatayo, O. C., Ayanda, J. T., Moyin‐Jesu, J., Isioye, A., & Popoola, A. A. (2021). A Geographical Analysis of the African COVID‐19 Paradox: Putting the Poverty‐as‐a‐Vaccine Hypothesis to the Test. *Earth Systems and Environment*, 5, 799–810.

56 Bozorgmehr, op. cit. note 9, p. 6.

57 Lakoff, A. (2017). *Unprepared: Global Health in a Time of Emergency*. University of California Press.

58 de Castañeda, R. R., Villers, J., Guzmán, C. A. F., Eslanloo, T., de Paula, N., Machalaba, C., et. al. (2023). One Health and planetary health research: Leveraging differences to grow together. *The Lancet Planetary Health*, *7*(2), e109‐e111.

59 Hunter D, J., & Dawson, A. (2011). Is there a need for global health ethics? For and against. In S. Benatar & G. Brock (Eds.), *Global Health and Global Health Ethics* (pp. 77‐88). Cambridge University Press, p. 88.

60 Van Batavia, J. P., Shukla, A. R., Joshi, R. S., & Reddy, P. P. (2018). Pediatric Urology and Global Health: Why Now and How to Build a Successful Global Outreach Program. *The Urologic Clinics of North America*, 45(4), 623–631.

61 Beaglehole & Bonita, op. cit. note 23, p. 3402.

62 Brandt A. M. (2013). How AIDS invented global health. *The New England Journal of Medicine*, *368*(23), 2149–2152.

63 Bozorgmehr, op. cit. note 9.

64 Kickbusch, I, (2002). Global Health ‐ A definition. Retrieved February 9, 2025, from https://www.ilonakickbusch.com/kickbusch-wAssets/docs/global-health.pdf

65 Rowson, M., Willott, C., Hughes, R., Maini, A., Martin, S., Miranda, J. J., Pollit, V., Smith, A., Wake, R., & Yudkin, J. S. (2012). Conceptualising global health: Theoretical issues and their relevance for teaching. *Globalization and Health*, 8, 36.

66 Bozorgmehr, op. cit, note 9, p. 6

67 King & Koski, op. cit. note 12.

68 Hunter & Dawson, op. cit. note 59.

69 King & Koski, op. cit. note 12.

70 Salm, et al., op. cit. note 20.

71 Squires, N. (2018, September 4). *Global health ‐ what it means and why PHE works globally*. UK Health Security Agency Blog. Retrieved August 24, 2024, from https://ukhsa.blog.gov.uk/2018/09/04/global-health-what-it-means-and-why-phe-works-globally

72 Brown & Moon, op. cit. note 43.

73 Yach, D., & Bettcher, D. (1998). The Globalization of Public Health, I: Threats and Opportunities. *American Journal of Public Health*, 88(5), 735–738.

74 Appiah, A. K. (2007). *Cosmopolitanism. Ethics in a World of Strangers*. Penguin Books.

75 Kleingeld, P., & Brown, E. (2019). Cosmopolitanism. In E. N. Zalta (Ed.), *The Stanford Encyclopedia of Philosophy*. The Metaphysics Research Lab.

76 Singer, P. (1972). Famine, affluence, and morality. *Philosophy and Public Affairs*, 1(3), 229‐243.

77 Horton, op. cit. note 22.

78 Peacock, S. J. (2022). Vaccine nationalism will persist: Global public goods need effective engagement of global citizens. *Globalization and Health*, 18(1), 14.

79 Pushkaran, et al., op. cit. note 17.

80 Ferguson & Caplan, op. cit. note 16.

81 Emanuel, E. J., Buchanan, A., Chan, S., Fabre, C., Halliday, D., Leland, R. J., et al. (2021). On the Ethics of Vaccine Nationalism: The Case for the Fair Priority for Residents Framework. *Ethics & International Affairs*, *35*(4), 543–562.

82 E.g. Boorse, C. (1977). Health as a theoretical concept. *Philosophy of Science*, 44(4), 542–573.

83 Nordenfelt, L. (1993.) On the relevance and importance of the notion of disease. *Theoretical Medicine*, 14(1), 15–26.

84 Cooper, R. (2002). Disease. *Studies in History and Philosophy of Biomedical Sciences*, 33(2), 263–282.

85 Barnes, E. (2023). *Health Problems: Philosophical Puzzles about the Nature of Health*. Oxford University Press.

86 Boorse, op. cit. note 82.

87 Cooper, op. cit. note 84.

88 Powell, R., & Scarffe, E. (2019). Rethinking “Disease”: A fresh diagnosis and a new philosophical treatment. *Journal of Medical Ethics*, 45, 579‐588.

89 Bickenbach, J. (2015). WHO's definition of health: Philosophical analysis. In T. Schramme & S. Edwards (Eds.), *Handbook of the Philosophy of Medicine* (pp. 961–974). Dordrecht: Springer Science.

90 Wakefield, J. (1992). The concept of mental disorder: On the boundary between biological facts and social values. *American Psychologist*, 47(3), 373–388.

91 Smart, B. (2022). Concepts of Health and Disease in Public Health, In S. Venkatapuran & A. Broadbent (Eds.), *The Routledge Handbook of Philosophy of Public Health* (pp. 53‐69). Routledge.

92 Amoretti, M. C., & Lalumera, E. (2022). Wherein is the concept of disease normative? From weak normativity to value‐conscious naturalism. *Medicine, Health Care, and Philosophy*, *25*(1), 47–60.

93 Kingma, E. (2007). What Is It to Be Healthy? *Analysis*, 67(2), 128–133.

94 Powell & Scarffe, op. cit. note 88.

95 Smart, op. cit. note 91, p. 56

96 Boorse C. (2014). A second rebuttal on health. *The Journal of Medicine and Philosophy*, 39(6), 683–724.

97 Salomon, J., Mathers, C., Chatterji, S., Sadana, R., Ustun, T. B., & Murray, C. J. L. (2003). Quantifying individual levels of health: Definitions, concepts, and measurement issues. In C. J. L. Murray & D. B. Evans (Eds.), *Health systems performance assessment debates, methods and empiricism* (pp. 301‐318*)*. World Health Organization, Geneva, p. 303.

98 King, L. S. (1954). What is Disease? *Philosophy of Science*, 21(3), 193–203.

99 Ereshefsky, M. (2009). Defining “health” and “disease.” *Studies in History and Philosophy of Biological and Biomedical Sciences*, 40(3), 221–227.

100 Nelson, J. L. (1995). Health and Disease as ‘Thick’ Concepts in Ecosystemic Contexts, *Environmental Values*, 4(4), 311–322.

101 Smart, op. cit. note 91.

102 Boorse, op. cit. note 82.

103 Smart, op. cit. note 91.

104 Wakefield, op. cit. note 90.

105 Wiland, E. (2022). What is Group Well‐Being? *Journal of Ethics and Social Philosophy*, 21(1).

106 Morar, N., & Skorburg, J. (2018). Bioethics and the hypothesis of extended health*. Kennedy Institute of Ethics Journal*, 28(3), 341–376, p. 361.

107 Smart, op. cit. note 91.

108 King & Koski, op. cit. note 12.

109 Smart, op. cit. note 91.

110 Wakefield, op. cit. note 90.

111 Gale, E. A. (2013). Is type 2 diabetes a category error? *Lancet*, 381(9881), 1956‐1957.

112 Thomas, C. C., & Philipson, L. H. (2015). Update on diabetes classification. *The Medical Clinics of North America*, *99*(1), 1–16.

113 Gill, G. V., Mbanya, J. C., Ramaiya, K. L., & Tesfaye, S. (2009). A sub‐Saharan African perspective of diabetes. *Diabetologia*, 52(1), 8–16.

114 Ibid:10.

115 Moran‐Thomas, A. (2019). *Traveling with Sugar: Chronicles of a Global Epidemic*. University of California Press.

116 Sarangarajan, R., & Ewuoso, C. (2024). Does the South African government have a duty to fund influenza vaccination of adults 65 years and older? *Frontiers in Public Health*, 12, 1303949.

117 Metz, T. (2010). Human dignity, capital punishment, and an African moral theory: Toward a new philosophy of human rights. *Journal of Human Rights*, 9(1), 81–99.

118 Nyamnjoh, F. B. (2015). Incompleteness: Frontier Africa and the Currency of Conviviality. *Journal of Asian and African Studies*, 52(3), 253–270.

119 Metz, op. cit. note 117.

120 Sarangarajan & Ewuoso, op. cit. note 116.

121 Engebretsen, E., & Heggen, K. (2015). Powerful Concepts in Global Health; Comment on “Knowledge, Moral Claims and the Exercise of Power in Global Health”. *International Journal of Health Policy and Management*, 4(2), 115‐117.

122 Barnett, M., & Duvall, R. (2005). Power in international politics. *International Organization*, *59*(1), 39‐75.

123 Shiffman J. (2014). Knowledge, moral claims and the exercise of power in global health. *International Journal of Health Policy and Management*, *3*(6), 297–299.

124 Lakoff, op. cit. note 57, p. 72

125 Ibid.

126 Pushkaran, et al., op. cit. note 17.

127 Osayomi et al., op. cit. note 55.

128 Bell, D., Brown, G. W., Oyibo, W. A., Ouédraogo, S., Tacheva, B., Barbaud, E., Kalk, A., Ridde, V., & Paul, E. (2023). COVAX ‐ Time to reconsider the strategy and its target. *Health Policy Open*, 4, 1000961.

129 Polu, S. L. (2012) *Infectious Disease in India, 1892‐1940: Policy‐Making and the Perception of Risk*. Palgrave Macmillan.

130 de Campos‐Rudinsky, T. C., Bosha, S. L., Wainstock, D., Sekalala, S., Venkatapuram, S., & Atuire, C. A. (2024). Decolonising global health: Why the new Pandemic Agreement should have included the principle of subsidiarity. *The Lancet Global Health*, *12*(7), e1200–e1203.

131 Hussain, et al., op. cit. note 14.

132 Táíwò, O. (2022). *Against Decolonisation: Taking African Agency Seriously*. Hurst Publishers.

133 Contractor, S. Q., & Dasgupta, J. (2022). Is decolonisation sufficient? *BMJ Global Health*, 7(12), e011564.

134 Anand, S. S., & Pai. M. (2023), Glocal is global: Reimagining the training of global health students in high‐income countries. *The Lancet Global Health*, 11(11), e1686–e1687.

135 Kickbusch, I. (1999). Global+ local= glocal public health. *Journal of Epidemiology and Community Health*, 53(8), 451–452.

